# Integrating Primary Care Services into a Rural Behavioral Health Facility in Northern Arizona: Perspectives of Healthcare Providers and Administrative Staff

**DOI:** 10.3390/healthcare13233050

**Published:** 2025-11-25

**Authors:** Jeffersson Santos, Amanda Acevedo-Morales, Lillian Jones, Carolyn Camplain, Stephanie Babbitt, Chesleigh N. Keene, Tara Bautista, Julie A. Baldwin

**Affiliations:** 1 Edson College of Nursing and Health Innovation, Arizona State University, Phoenix, AZ 85004, USA; 2 Center for Community Health and Engaged Research, Northern Arizona University, Flagstaff, AZ 86011, USA; 3 School of Nursing, Universidade Federal de Sergipe, Aracaju 49015, SE, Brazil; 4 Department of Clinical Psychology, Northern Arizona University, Flagstaff, AZ 86011, USA; 5 Department of Applied Health Science, School of Public Health-Bloomington, Indiana University, Bloomington, IN 47407, USA; 6 Community Health Advisor, Flagstaff, AZ 86001, USA; 7 Kauffman and Associates, Incorporated, Flagstaff, AZ 75215, USA; 8 College of Education and Human Sciences, University of New Mexico, Albuquerque, NM 87106, USA

**Keywords:** rural health, behavioral health, primary care, substance use disorders, integrated care

## Abstract

**Background/Objectives:** Integrating behavioral health and primary care services is a national public health priority in the US, especially in underserved settings like northern Arizona. This healthcare delivery model is crucial to meet the mental and physical health needs of people with SU/SUDs, particularly those belonging to culturally diverse populations. In collaboration with a behavioral health center in northern Arizona, the current study aimed to assess the perspectives of providers and administrative staff on the implementation of integrated primary care (IPC) services for people with SU/SUDs. **Methods**: In February 2023, twelve healthcare providers and administrative staff from diverse educational backgrounds were recruited using purposive sampling to capture a range of perspectives on IPC implementation at the behavioral health center. Participants completed individual, semi-structured interviews conducted via Zoom, which were audio recorded and lasted approximately 30 min. The interview recordings were transcribed verbatim using Trint Software, and analyzed on Google Docs using applied thematic analysis. Two researchers coded the transcripts, iteratively developing and refining themes through multiple cycles of review and team discussions. Additional team members provided feedback and verified the themes, with consensus reached through collaborative meetings. This rigorous, iterative approach ensured the reliability and validity of the final thematic framework. **Results**: We found that IPC supports SU/SUDs recovery by providing holistic care that integrates medical, mental health, and addiction services while addressing social and co-occurring needs. It fosters an empathetic environment where clients do not need to repeatedly disclose their SU/SUDs, improves access to preventive care, and offers continuous support and education. Implementation barriers included workforce shortages, limited internal communication, and insufficient interdisciplinary training. Gaps in culturally centered care were identified, including reliance on Western models, limited representation of Native American and sexual and gender minority staff, and inconsistent use of inclusive practices such as pronouns, traditional healing, and trauma-informed approaches. Additionally, community partnerships with multisectoral organizations help clients access supportive resources beyond the facility, including vision care, clothing, and dental services. **Conclusions**: The implementation of IPC was seen as important to support the behavioral health center in northern Arizona to foster an empathetic environment where clients with SU/SUDs can have their mental, physical, and social needs addressed, either within the facility or through community partnerships, thereby supporting their recovery. However, progress is hindered by barriers such as workforce shortages, limited internal communication, and insufficient interdisciplinary care training. Additionally, despite regular cultural competency training, gaps remain in culturally centered care for underserved populations, particularly Native American and sexual and gender minority clients.

## 1. Introduction

The integration of primary care within behavioral health settings, known as integrated primary care (IPC), is a healthcare delivery approach that can address both the physical and behavioral health needs of people with substance use or substance use disorders (SU/SUDs) through coordinated, interdisciplinary care [1]. Assessing newly implemented IPC services is important to ensure that they are optimally serving clients with SU/SUDs, individuals who experience a two-fold challenge: heightened risk for adverse physical health outcomes linked to SU/SUDs, alongside heightened barriers to primary care services due to factors like limited provider training to care for people with SU/SUDs, stigma, and financial constraints [2,3]. Evaluations of IPC services are particularly important in rural areas of the United States (US), such as Northern Arizona, an underserved region that spans around 66,000 square miles (≈170,939 square kilometers) comprising small towns with populations under 100,000, interspersed with federally protected forests, desert landscapes, and Native American Reservations [4,5]. Advancing research on IPC is considered a regional priority in Northern Arizona to help tackle health disparities that stem from chronic shortages of both behavioral health and primary care services, long distances to available healthcare services, and limited transportation options [4]. By offering primary care services within behavioral health centers in northern Arizona, IPC can support SU/SUDs recovery for residents in this underserved region by promoting convenient access to a continuum of health promotion services that include health education, diagnosis of SU/SUDs-related physical health conditions, and management of chronic health issues [6].

Assessments of IPC services should also consider that integrated care models are a powerful outlet to address the social determinants of health of people with SU/SUDs [7]. The Social Determinants of Health (SDHs) Framework underscores the critical role of societal factors in shaping people’s health outcomes [8]. These determinants are categorized into five core domains: economic stability, access to and quality of education, healthcare quality and access, neighborhood and built environment, and social and community context [8]. The healthcare quality and access domain is of particular importance to contextualize the importance of this study, as the availability of IPC services that are responsive to clients with SU/SUDs is a key factor to ensure these individuals can access needed comprehensive care. However, IPC services alone do not have the capacity to address other factors across the SDHs domains that influence the health of clients with SU/SUDs, such as homelessness, food instability, social isolation, and limitation to health services (e.g., dental and vision care) not commonly available in behavioral health centers, problems that are common in northern Arizona [4]. The integration of care services like IPC is a way to bring together community resources to promote the overall well-being of people with SU/SUDs, which can be achieved through partnerships fostered by the interdisciplinary care team with local organizations that target social determinants of health [9,10]. Therefore, assessments of IPC services in northern Arizona are collaborating with local resources and communities to effectively address these social determinants of health.

Assessments of IPC services in rural settings, such as northern Arizona, should consider how these programs provide care that is responsive to the cultural backgrounds of people with SU/SUDs from diverse backgrounds [11]. Native Americans, Latino populations, and lesbian, gay, bisexual, transgender, and queer (LGBTQ+) people, priority populations for public health research in northern Arizona, experience an increased risk of substance use as a coping mechanism in response to minority stressors stemming from systemic oppression related to their identities [4,12,13,14,15]. A major barrier to care-seeking behaviors for these populations is the limited capacity of health facilities to provide culturally centered care, care that respects and aligns with the specific needs and contexts of diverse populations, particularly for addressing SU/SUDs or other related issues (e.g., HIV testing after risky sex while intoxicated) [4,16,17,18,19]. Therefore, efforts to advance IPC in northern Arizona must consider culture as a central dynamic within healthcare systems to promote trust and engagement to care among minoritized populations with SU/SUDs, as they might have limited available healthcare options to turn to when needed [18].

The successful implementation of novel models of care, such as IPC, requires more than just starting new services; it demands a transformation in the healthcare facility’s organizational culture, enabling effective collaboration between providers and administrative staff. Such collaboration is key for the success of the implementation process and sustainability of the new healthcare services. By gaining insight into the perspectives of staff engaged in providing and implementing IPC in rural settings, involved parties (e.g., facility directors, funding organizations, policymakers) can identify critical barriers and facilitators for its successful implementation. This understanding is crucial for ensuring that IPC effectively meets the health needs of rural residents with SU/SUDs, especially those belonging to culturally diverse backgrounds [9].

Although advancing IPC in rural regions is recognized as a national priority, limited research has documented how these services function in practice or the factors that support or hinder their implementation [4,5]. Less attention has been paid to the delivery of culturally centered care within IPC services to support diverse populations with SU/SUDs [20]. In northern Arizona, prior research has highlighted a need to better understand how IPC services are supporting people with SU/SUDs and whether these services are responsive to the cultural and social contexts of the populations served [20]. Previous qualitative IPC assessments have also emphasized the importance of examining the perspectives of professionals in IPC services to identify barriers and facilitators to IPC delivery, which can inform targeted strategies for strengthening care integration [6]. To address this research gap, this study aimed to assess the perspectives of healthcare professionals and administrative staff at a behavioral health center in northern Arizona regarding their experiences with the implementation and delivery of IPC services for people with SU/SUDs.

## 2. Methods

### 2.1. Study Setting

The study took place at a community behavioral health center serving a northern Arizona population, including 12 Native American Nations, Latino communities, and LGBTQ+ people. The facility offers a variety of outpatient and residential SU/SUD treatment and emergency psychiatric services. State-licensed IPC services started in 2021 with comprehensive health assessments, physical examinations, referrals to specialized care, medication management, and health education. The integrated services support individualized care plans for clients. The IPC clinic is staffed by a full-time Family Nurse Practitioner (MSN, FNP-BC, APRN) and a full-time Medical Assistant who are both on-site Monday through Friday, 8:00 a.m. to 5:00 p.m. The clinic serves members from the psychiatric hospital, two residential treatment units, and outpatient programs. All major commercial insurance plans and all Medicaid plans are accepted. While services are available only during standard business hours, this structure aligns with the overall integration model on the behavioral health campus, where medical and behavioral health staff coordinate care directly. All providers and staff at the facility receive annual cultural competency training that includes content on LGBTQ+ people, Native Americans, and Latino communities. This training is supplemented by community partnerships and bilingual staff who facilitate communication and outreach with patients and families who speak Spanish or Native American languages.

### 2.2. Study Design

This was a qualitative study conducted through semi-structured individual interviews and a community-engaged approach. The study was developed through the Culturally Centered Addictions Research Training, a graduate certificate program focused on training doctoral students in health-related fields and practicing clinicians in SU/SUDs research, with an emphasis on minoritized populations living in rural areas of the US [21]. As part of this training, graduate students from health-related programs worked in pairs under the supervision of research professors. Students and professors were connected with representatives from behavioral health organizations in northern Arizona. Facility representatives share priorities for their organizations, providing students the opportunity to partner with facilities whose priorities align with their research interests [22].

Representatives from the behavioral health center that partnered with the students for this study, including members of the facility director and quality improvement team, identified several research topics aimed at improving care delivery. Research on the integration of primary care services into the behavioral health facility was identified as one of the priorities, aligning with the research interests of students and their professors. Such a research area is also considered a regional priority in northern Arizona to enhance support for people with SU/SUDs [4].

The independence of the research team was a cornerstone of this study. All aspects of the study were conducted by students under the supervision of professors. Facility representatives’ roles were limited to ensuring access to the facility and providing contextual input for this manuscript. Both strengths and opportunities for improvement of the behavioral health center are addressed in this paper, which underscores the independence of the research team while maintaining alignment with community needs.

### 2.3. Study Participants and Data Collection

Participant recruitment was conducted through purposive sampling by the student researchers. Potential participants were recruited through a secure message sent by the students through the behavioral health centers’ institutional email in January 2023. To be included in the study, individuals had to (1) be 18 years of age or older, (2) be currently working as a healthcare professional or administrative staff at the facility. Twelve professionals with backgrounds in nursing, counseling, case management, and medical assistance met the inclusion criteria and enrolled in the study.

A member of the research team, a student enrolled in Northern Arizona University’s Interdisciplinary Health PhD Program and Culturally Centered Addictions Research Training Graduate Certificate, who had no prior relationship with the participants, conducted audio-recorded interviews with participants via Zoom in February 2023. We opted to conduct interviews via Zoom to promote participant confidentiality and convenience, which enabled participation in the study from any preferred location and allowed flexible scheduling when needed. The interviews, held individually in either a private or workplace setting, depending on participant preference, lasted approximately 30 min each. The semi-structured interviews followed the guide described in Table 1. Interviews continued until thematic saturation was achieved. Saturation was assessed iteratively throughout data collection, and by the time 12 participants had been recruited, no new themes or information were emerging. Participants received a $30 Tango gift card as compensation.

This study was approved by Northern Arizona University’s Institutional Review Board (#1972881-1), which classified it as minimal-risk research. All participants received a written consent form via email and provided verbal consent prior to the interview, which aimed to promote convenience for participants while ensuring full information about all aspects of the study. Participants were assured that their participation was voluntary and confidential, that there were no right or wrong answers, and that they were free to share their honest opinions. Audio recordings and transcripts were stored on a secure drive (ADAMS server) to ensure data security.

### 2.4. Data Analysis

The data were analyzed using applied thematic analysis. The interviews were audio recorded and transcribed verbatim using Trint Software (trint.com, 2022) and manually analyzed for themes and codes using Google Docs. A single researcher (AA) initially reviewed each transcript alongside the corresponding audio recordings and organized the twelve interviews, generating a preliminary list of codes based on recurring words and patterns of experience. Another team member (JS) independently reviewed the transcripts, also verifying them against the audio recordings, and provided feedback on the identified codes, themes, and subthemes. These codes were iteratively interpreted and grouped into themes through two cycles of analysis: the first completed in October 2023 and the second in December 2023.

To refine the thematic structure, two thematic review meetings were conducted in December 2023, attended by AA, JS, TB, and CK. In the first meeting, emergent themes identified by AA and JS were reviewed. During this process, TB and the CK identified two themes requiring further review for consensus and two themes that necessitated a second thematic analysis of quotes to determine whether sufficient data supported their inclusion. As a result of this discussion, one theme was modified to “barriers to care integration” and two subthemes were identified and integrated into the coding framework.

A second thematic review meeting was held in December 2023, with JS, CK, TB, and AA in attendance. Key discussion points from this meeting included refinement of the coding strategy by JS and AA. Specific adjustments made during this meeting included verifying the inclusion of partnership-related themes, adding additional quotes under new themes, and verifying the relevance of the topic of training under the newly identified partnership theme, with discussion around whether partnerships and referrals should be distinct categories.

Following these discussions, JS and AA concluded their analysis and a third team member (JB) conducted an independent review in May 2024, confirming the validity of the refined themes and subthemes. Any remaining disagreements were addressed through a group discussion involving all team members, leading to the finalization of a comprehensive thematic structure by the end of May 2024. This iterative, collaborative process ensured a rigorous and systematic approach to the qualitative analysis, enhancing the reliability and depth of thematic interpretations.

## 3. Results

A total of five themes and eight sub-themes were ultimately identified in the study. The themes were (1) IPC as a comprehensive approach to SU/SUDs care, (2) barriers in the implementation of IPC in rural Arizona, (3) benefits of integrated care in rural Arizona, (4) the current state of culturally centered practices in service delivery, (5) The role of community partnerships and referrals in supporting the mental, physical, and social needs of people with SU/SUDs in rural Arizona.

### 3.1. Theme 1: IPC as a Comprehensive Approach to SU/SUDs Care

Participants highlighted IPC as essential for addressing the complex needs of individuals seeking SU/SUD recovery. One participant emphasized the importance of a streamlined care system that holistically integrates medical, mental health, and addiction services.

*“I think of it [IPC] as a streamlined care system where a person who is driving into recovery and sobriety is going to have their medical health needs met, as well as mental health needs, addiction services is where basically everything is coming together for the person.”*

Additionally, participants discussed that IPC is an ideal healthcare model because of the strong connection between mental and physical health, as well as the broader psychosocial stressors that contribute to substance use.

*“In an ideal world, both the primary care provider and the addiction services providers are working together to meet the holistic needs of the client.”*

*“Mind and body are connected, and the impact of substances on the body is one of the biggest negatives.”*

Participants also noted that IPC helps understand clients with SU/SUDs within their social and environmental contexts, rather than in isolation.

*“We can be very compassionate and understand things from like a person in the environment as a whole…and recognize all the psychosocial stresses that they’re dealing”*

Another participant emphasized that IPC allowed facilities to be seen as offering a “full spectrum” of services, highlighting the need for comprehensive care in recovery settings. IPC was also viewed as vital for addressing other mental health concerns linked to SU/SUDs, including depression and anxiety.

*“[IPC is important] To recognize us as a full spectrum facility that they [people with SU/SUDs] can get their services out.”*

*“I think that it [IPC] would be care from a holistic approach. So, helping people not only with substance use or abuse, but also making sure the different mental health aspects outside of that are dealt with as well, like depression, anxiety, even things like homelessness or racial barriers.”*

### 3.2. Theme 2: Barriers in the Implementation of IPC in Rural Arizona

#### 3.2.1. Subtheme 1: Challenges in Retaining and Recruiting Providers in Rural Arizona

Provider retention and recruitment were identified as significant barriers to IPC implementation in rural Arizona.

*“I think right now we have a hard time hiring and maintaining and keeping staff in [city]. I would like to see more team building and that sort of thing to try to have more employee retention.”*

*“I mean, you know, there’s limited providers in [city] because we’re kind of semi-rural.”*

Many facilities struggle to attract and retain qualified providers, leading to staffing shortages that disrupt continuity of care for individuals with SU/SUDs. One participant emphasized that, beyond hiring additional staff, providers must feel valued and heard within the system. Creating a supportive work environment where provider input is acknowledged was seen as essential for long-term retention and effective IPC delivery.

*“I think that if we hired more people with the specific intention of making sure that everyone’s voice is heard, I think that that would be helpful.”*

#### 3.2.2. Subtheme 2: Limited Internal Communication Within the Facility

Communication breakdowns were noted as a persistent challenge in IPC, affecting coordination between healthcare providers and clients. To address this, one participant described using medical advocacy to bridge gaps and ensure clients receive the care they need. This advocacy streamlines care and helps clients feel heard and supported.

*“I do have a couple clients that I provide medical advocacy for just to ensure that they’re getting the care that they feel like they need. Sometimes it’s a breakdown of communication between them and the provider, but when there’s a third-party monitoring what’s going on, then the care is more streamlined…”*

While advocacy plays a critical role, participants suggested that more structured communication among providers could improve care coordination. One participant proposed regular meetings between prescribers and therapists to align treatment plans and promote a more collaborative approach. Another provider reflected on cases where they had minimal interaction with primary care providers, noting that care often remains compartmentalized rather than fully integrated.

*“If we had a monthly meeting between prescribers and therapists, for instance, to talk about the clients and maybe get on the same page as far as nutrition and supplements as well as medications.”*

*“I personally have not been involved in the case where we’re working closely with the primary care providers to, you know, coordinate things or it’s always kind of been very compartmentalized in my experience.”*

The limited internal communication between case managers and primary care providers further reinforces silos. Two participants succinctly described the ongoing issue of limited information exchange, emphasizing the need for a more interconnected system.

*“There’s generally not a lot of reciprocal communication between us (case manager and primary care provider) going on.”*

*“I’ll be honest, I feel like there are a lot of issues with communication between departments.”*

#### 3.2.3. Subtheme 3: Limited Staff Training and Education About Best Practices of Integrated Care

Participants emphasized the need for improved education and training for clinical staff to bridge knowledge gaps and ensure consistency in IPC delivery. One participant succinctly captured this overarching challenge.

*“I think the biggest barrier [to integrated care] is just the lack of education.”*

Limited training was identified as a barrier to effectively managing complex aspects of care, such as understanding the mental health implications of medication side effects. As one participant elaborated, a deeper awareness of these interactions is essential for providing comprehensive care.

*“Probably more education for the staff. Understanding that there’s so many different side effects and things like that of medication and how it affects the mental aspect.”*

Another participant highlighted the need for standardized training or policies to align providers across different roles. Establishing clear guidelines could help define the scope of practice and responsibilities, ultimately strengthening the integration of care.

*“I think there needs to be training or a policy or something where providers are all on the same page on the services they provide and their boundaries.”*

### 3.3. Theme 3: Benefits of Integrating Primary Care into a Rural Behavioral Health Center

#### 3.3.1. Subtheme 1: Reduction in Stigmatizing Views About Access to Behavioral Healthcare Facilities

Participants identified IPC as a crucial tool for reducing stigma associated with behavioral health and SU/SUDs treatment. By consolidating multiple services in one location, IPC normalizes treatment and fosters a supportive environment for individuals seeking care. One participant emphasized how this approach helps address feelings of shame and low self-worth often experienced by individuals with SU/SUDs.

*“A lot of our members and nonmembers that are just trying to get into rehab or mental health care have a lot of shame when it comes to addiction. They have a lot of self-worth issues and there’s a lot of things going on with their bodies and they’re disconnected from their bodies. It’s nice to know that somebody is willing to see them and to take care of them. It’s a caring environment. It’s important with addiction is that they feel that they’re worthy.”*

Another participant highlighted that IPC settings create a judgment-free space, reducing the burden of repeated self-disclosure. By eliminating the need to constantly revisit their addiction history, individuals can engage in care more comfortably and without fear of stigma.

*“With addiction, there’s a lot of people feel like they can’t go to a regular provider office for the fear of being judged. But with integrated care at the [behavioral health center], they already have established that their addiction and what they’re struggling with and we are working with them to get them stable and sober so that where they don’t have to disclose that information.”*

#### 3.3.2. Subtheme 2: Facilitating Access to Preventive Care Among Clients with SU/SUDs

Participants highlighted preventive services as a key benefit of IPC for individuals with SU/SUDs. Given the interconnected nature of physical and mental health, as well as systemic factors contributing to adverse health outcomes, targeted approaches are essential for promoting recovery and preventing further complications. Addressing these dimensions holistically ensures that individuals receive comprehensive care to support their overall well-being.

*“People’s bodies have been damaged through substance use. And even prior to the substance use, they may have had either nutritional deficiencies or issues that led to addiction in the first place or mental health issues. So, it’s all connected to physical and mental health.”*

*“The longer that they have this addiction, the more prone they are susceptible to developing other ailments, to other illnesses, and other complications that they might not be aware of. But until they get those services then they’ll be educated on if anything further has developed.”*

IPC provides continuous support and education, helping individuals navigate the recovery process more effectively. By offering services such as medically assisted detox, wellness care, and regular screenings, IPC plays a crucial role in preventing additional health complications and supporting long-term recovery.

*“[IPC] is important to prevent for any individual, but for folks that are receiving treatment for addiction and going through that recovery process, there’s a lot there…mentally, physically. They need as much support and treatment as much as we can provide…First they need to perhaps go through a medically assisted detox. Then it goes on to finding that wellness and care throughout the recovery. Now that they’re feeling aches and pains. It’s offering that service to them throughout their care, and as much as they accept it.”*

One participant emphasized the need to further strengthen IPC’s impact on preventive care by integrating behavioral health consultants into primary care units. This addition was suggested as a strategy to enhance screening processes, facilitate early interventions, and provide tailored support for individuals with SU/SUDs.

*“I think it would be great if [IPC] had a behavioral health consultant, which would be a social worker that is dedicated to the primary care unit…that role can provide screening for brief interventions.”*

### 3.4. Theme 4: The Current State of Culturally Centered Practices in Service Delivery

#### 3.4.1. Subtheme 1: Healthcare Providers Must Be Mindful of Clients’ Diverse Cultural Backgrounds

Participants highlighted that culturally centered care within IPC involves being mindful of the diverse identities and experiences of clients with SU/SUDs. They stressed the need to acknowledge how cultural backgrounds shape health beliefs and behaviors among people with SU/SUDs while cautioning against assumptions based solely on race. Additionally, participants noted that providers must take the time to understand each client’s unique cultural identity.

*“Each patient brings their own culture. The idea is to just get to know your patient and know your client really well. You can’t automatically assume that someone is a certain culture just because that’s their race.”*

*“Whatever populations are being served in the organizations, city, county, state, whoever you’re going to encounter, you have to have some cultural fluency with understanding who you’re talking to so that they can feel, heard and seen and understood and be getting appropriate care.”*

Participants stressed the importance of providers understanding the communities they serve, including community demographics and substance use patterns, as structural factors can greatly influence how people perceive SU/SUDs. This need was reported to be especially critical due to challenges related to immigration in rural Arizona, particularly undocumented immigration, as individuals without documents may avoid seeking care due to fear of deportation.

*“A person’s culture has everything to do with the way that they approach their physical and mental health, how comfortable they are talking about those things, and the prevalence of addiction or likelihood of addiction in their particular community. So, the providers who are treating that person have to be able to understand and relate and care for them in a way that is appropriate for their culture.”*

*“You have to understand there, you’re working a lot with a population of Mexican immigrants, both documented and undocumented, who may be terrified to seek care because they’re worried that they’re going to be deported if they’re undocumented.”*

Participants emphasized that effective IPC requires awareness of culturally specific communication styles. They noted that behaviors perceived as inappropriate in some settings may be considered normal in others, underscoring the importance of contextual interpretation. Additionally, participants highlighted how cultural perspectives on mental health and addiction can influence healthcare interactions. Some communities, they explained, prefer to address behavioral health within families rather than seeking professional care, reinforcing the need for providers to approach these differences with cultural sensitivity.

*“The big thing is understanding where they’re coming from and that they may say something to you that [might be perceived as] rude, but you have to know that they may not even know they’re saying something rude. They may think that’s actually a very nice thing to say. So, in culture, we’re making sure that the staff are culturally aware…can also help with positive psychological and physical health.”*

*“Some communities are more comfortable talking amongst themselves about mental health and addiction issues. And in other cultures, that’s still a bit taboo or it’s not talked about as much, or you’re supposed to take care of it in the family. I had one client that was saying ‘my mom doesn’t understand why I’m talking to a stranger instead of just keeping it in the family’. Meaning me… like I’m a stranger, right? I’m a therapist.”*

Participants stressed the need for trauma-informed care in IPC to address the cumulative stresses people with SU/SUDs face, such as systemic inequalities and risks for sexually transmitted infections. A compassionate, trauma-informed approach was considered vital for client engagement. Religious and spiritual beliefs also significantly shape health decisions. Providers must recognize these influences and navigate potential conflicts related to spirituality, ensuring inclusive care.

*“Trauma-informed care training [is needed] so that we can be very compassionate and understand things from a person in the environment as a whole rather than just this isolated individual …maybe screening for high-risk behavior depending on what culture they’re in and the substances that they’re using for, sexually transmitted diseases, those kinds of things.”*

*“If it’s a different kind of a faith-based things like Christianity or Mormonism, I think incorporating that as well is important (e.g., the prayer or the supports that a person might get through their church, congregation, community, or their pastor or preacher)”*

*“We had some clients that were very religious, devout Christians. They had a problem with another client that practiced Tarot. We had to constantly talk with them and get advice on how to best help these clients and inform the other clients like, ‘hey, this is their perspective.’ They see this as demonic, but is it really a demonic type thing?*

#### 3.4.2. Subtheme 2: Honoring Native Americans’ Beliefs and Practices

Participants emphasized that culturally centered care for Native Americans must address mental health stigma, particularly around suicide and SUDs, which are rarely discussed openly. They noted that distrust of external healthcare systems, especially government-funded programs, limits access to care.

*“It is taboo in Native American culture talking about suicide or death…It can be very difficult for Native Americans to feel comfortable with it…The rule is kind of you just don’t talk about it.”*

*“I’m Native American and Native American population is very big to me because of the lack of resources that are available on the reservation or having the trust to go into a government funded program. There’s a disconnect between Native American consumers not feeling like they’re going to get the same level of care as if their case manager was not Native American.”*

Adapting assessment tools used in healthcare to language more aligned with Native American cultures was seen as an approach to promote enhanced communication about mental health problems.

*“I think if we were able to adapt these widely used things like the Columbia Risk Assessment into a more culturally sensitive way, such as instead of asking, ‘have you ever had suicidal thoughts?’, instead maybe asking, ‘do you ever struggle with living in your day to day life?’ So instead of approaching it from the aspect of exclusively suicide, which they [Native Americans] might have a bad reaction to, it can instead approach it from a different angle.”*

Understanding the historical context of addiction is essential for quality care, yet often only Native American or similarly minoritized providers have this knowledge. Current service delivery practices were reported to be largely grounded in Western practices, with spiritual practices integrated only when requested. Participants stressed that Native Americans must advocate for traditional healing, which should be part of integrated care.

*“When we had the colonizers come in with the alcohol and all their cultural beliefs, the natives got pushed out…The providers, unless they are of culture, they don’t understand that. They just ‘all they just got an alcohol problem’. But why do you think that is? They’re not offered the same opportunities as people that look like you. It makes it easier for them to fall into those addictions.”*

*“We have a lot of clients who are Native American and I haven’t seen that their care is any different... I have had clients who’ve asked for things like access to a traditional practitioner, but they have to request that and even know that it’s available for them. So I feel like it’s a deficit on our part.”*

Proactively engaging clients about culturally centered practices is crucial to ensure awareness of service availability, along with hiring Native American providers to foster trust and address community needs, such as language barriers. Staff with shared lived experiences further ensure culturally responsive care.

*“I know a lot of Native Americans ceremonies and the families will request we incorporate that. So at the get go, encouraging the members to [request ceremonies] is important. So we have to pay attention to that.”*

*“Because we’re a border town to the reservation, [it is important to] have a consultant who trains providers, care managers, therapists, everybody across the board about cultural competency and the mistrust that comes from people from the reservation trying to seek services.”*

*“Having people who have the same lived experience has been helpful. One of the case managers speaks Navajo and also has experience being a recovered addict. That specific experience where he has the same cultural background and he’s also able to bridge the gap between languages [is important]… as many times English is not going to be someone’s first language if they grew up on the reservation or in a Native American family.”*

#### 3.4.3. Subtheme 3: Culturally Centered Care for LGBTQ+ People with SU/SUDs

The LGBTQ+ health training offered at the facility was seen as essential for helping providers understand the unique challenges this population faces and the importance of ensuring affirming care delivery, including recognizing how LGBTQ+-phobia and family rejection can contribute to stress-related coping behaviors and increase vulnerability to SU/SUDs. It was noted that LGBTQ+-affirming care includes clinical and administrative practices that ensure inclusive processes during intake, charting, and insurance documentation, which can prevent situations such as misgendering and other forms of identity invalidation.

*“We do frequent training on the LGBTQ community… understanding that these people don’t have the same experiences as us. They frequently relapse because maybe they grew up in a very homophobic environment. Some maybe turn to alcohol to help cope with the distress of being disowned by their family.”*

*“For the LGBT population, I think a big piece [of culturally centered care] is education. Doctors aren’t always aware of the intricacies that come with things like preferred names and preferred pronouns. What they see on a chart is kind of all they get ahead of time. So they might walk into a situation [e.g., misgendering] without knowing. If we can start the conversation at intake or even with insurance, then I think it will provide better care throughout the whole process.”*

Additionally, it was noted that providers must uphold the ethical standards of their profession by prioritizing the needs of their LGBTQ+ clients and setting aside personal beliefs. Another participant emphasized the importance of understanding LGBTQ+ substance use patterns, highlighting the prevalence of methamphetamine and cocaine among LGBTQ+ clients and in their clinical experience and the need for providers to tailor care accordingly.

*“I’ve seen a large number of members who are from the LGBTQ population, and I think it’s just being respectful…especially with people who identify differently from how they’re presenting. It’s very important to use the proper pronouns. I know that sometimes it can be an issue. Some people say it’s harder to do it. You just have to be mindful of what you’re doing and not worry about your own values and your own thoughts and opinions and be mindful of how others are presenting.”*

*“We know, for example, that people in the LGBTQ community, there can be a higher prevalence of recreational methamphetamine or cocaine in different areas, and that’s often not thought of as a problem. It’s like, I’m fine. I just like to have fun. You have to understand who you’re speaking with.”*

Despite the availability of regular LGBTQ+ health training sessions, gaps in LGBTQ+-friendly care persist. The concept of culturally centered care for LGBTQ+ individuals may not always be fully realized, but there is widespread recognition of its importance in creating an environment where these clients feel comfortable expressing their health needs.

*“We’re not doing culturally appropriate care. I’m an indigenous person and part of the LGBTQ+ community, and I don’t see that unless a case manager pushes for it.”*

*“I don’t know exactly what that [LGBTQ-centered care] looks like all the time. But I think being accepted is the number one thing and treating people with respect and dignity. Being in this business as long as I have, I cannot make people feel one way or another, but I can help them to feel more comfortable and feel part of the treatment and ask for what they need in their treatment.”*

Other barriers to LGBTQ+-friendly care included limited promotion of LGBTQ+ support services and inconsistent attention to clients’ pronouns.

*“We mess up everybody’s pronouns and I’m working on it. And part of it is just being more mindful and looking at the chart a little closer. That would be very validating to a lot of people that are transitioning.”*

*“We do have an LGBTQ plus support group, but it’s something we tell clients about if they ask about it. We don’t really have literature that we make available, like in the lobby.”*

### 3.5. Theme 5: The Role of Community Partnerships and Referrals in Supporting the Mental, Physical, and Social Needs of People with SU/SUDs in Rural Arizona

Participants highlighted the active partnerships with various rural community organizations that complement the facility’s integrated care services. By leveraging these partnerships, the center ensures clients have access to diverse resources and specialized services that go beyond its own offerings, effectively addressing their physical, mental, and social needs.

*“We also are in coordination with other agencies that provide things that we don’t provide.”*

Specific examples include partnerships addressing unique client needs, such as vision care, clothing, transportation, shelter, dental health, durable medical equipment, and food access.

*“We refer people out to like other community resources such as [local healthcare facility A] which helps them with groups that support transportation or medical appointments.”*

*“You know, they might need glasses. We work with the [local organization] to get individual glasses. We work with a thrift store to get people’s clothing. [Local healthcare facility A] has a dental program and we refer and get referrals from the dental program, which has a lot to do with heroin and can really affect your dental health.”*

*“We have so many individual organizations just in [city] alone, and it has primarily to do with resources for shelter, housing, vocational, and for continued recovery.”*

Eligible patients can access pharmacy services at a Native American health center, with their prescription costs covered by Native American health insurance.

*“We also work with [local healthcare facility B], which is an indigenous health center that does a lot of work with pharmacy work and things like that. So a lot of clients who are Native American will get their medications through that pharmacy because they work a lot better with American Indian Health Plan, which is the insurance used on the [indigenous] reservation and things like that.”*

Another important collaboration is with a local organization that provides peer support services. These services are run by individuals who have gone through SU/SUD recovery, ensuring that clients receive additional, empathetic, and relatable guidance throughout their recovery journey.

*“We also use [local organization C], which is more for a peer support aspect. And it’s run by. Former substance abuse people who are now living in sobriety. So there’s not really good peer support and lived experience kind of thing.”*

The facility also serves as a key support resource during public health emergencies, such as suicides in a local school and wildfires. The facility leads community outreach, coordinates with the Red Cross, and helps secure housing, clothing, and hygiene supplies for those affected, showing its commitment to addressing both immediate and long-term community needs.

*“There’s a lot of integration going on. The center does a good job reaching out to the community. When there’s been a disaster, the [facility] has been able to reach out to the community and provide additional support. When there was an episode of suicides that occurred at the junior high and I think a teacher passed away as well…we were able to go into the [school] and provide support. We’ve had a couple of fires that were really severe and we’ve been able to open up to the Red Cross and to the county and get housing and clothing, and hygiene supplies for people.”*

The behavioral health center also maintains strong collaboration with the criminal justice system. This close relationship was reported as valuable, as probation officers often refer clients with substance use-related charges to the behavioral health center, ensuring that they engage with SU/SUDs treatment services.

*“We get quite a few clients who got substance related charges and their probation officer wants them to engage. We have good communication with those folks.”*

## 4. Discussion

Advancing IPC in rural settings of the US, like northern Arizona, is a public health priority aimed at addressing disparities in healthcare access and health outcomes among people with SU/SUDs, particularly those from diverse backgrounds. In this study, we examined the experiences of healthcare professionals and administrative staff about the implementation and delivery of IPC services at a behavioral health center in northern Arizona. Participants viewed IPC as a comprehensive approach to effectively address the mental and physical health needs of individuals with SU/SUDs in rural areas of the US. Perceived benefits of IPC included a reduction in stigma related to healthcare access among people with SU/SUDs and engagement in preventive care services. However, barriers to implementing and sustaining IPC were identified, including difficulties in recruiting and retaining healthcare providers, limited internal communication, and limited staff training on best practices for behavioral health and primary care integration. Participants emphasized the relevant culturally centered practices that must be considered in IPC, including mindfulness of clients’ diverse cultural backgrounds, honoring Native American beliefs and practices, and delivering tailored care to LGBTQ+ individuals. Additionally, participants noted that maintaining partnerships with community organizations is crucial for ensuring that clients with SU/SUDs have access to needed resources (e.g., housing, food, healthcare) not available at the facility.

This study supports findings from a previous study conducted with clients from the same behavioral health center, which demonstrated that IPC facilitated access to preventive services [6]. Through IPC, clients were able to receive timely diagnoses of physical health conditions related to SU/SUDs and gain valuable education on healthy lifestyle practices. These efforts not only addressed immediate health concerns but also empowered clients to take proactive steps toward improving their overall well-being [6].

Similar to other studies, we identified difficulty in recruiting and retaining providers as a barrier to implementing and sustaining IPC services [23,24]. The shortage of healthcare professionals to serve people with SU/SUDs in rural areas is a long-standing public health challenge. To address this issue, IPC services may benefit from targeted recruitment strategies that prioritize providers with prior experience in caring for underserved populations [25]. Higher education institutions can also play a vital role in tackling the healthcare shortage in the US by prioritizing admission and financial support for students interested in practicing in rural settings. Both healthcare facilities and higher education systems can prioritize recruiting, educating, and retaining individuals from the culturally diverse rural communities they serve. Additionally, medical societies, hospitals, the state and federal government should advocate for policies that facilitate the immigration process of international healthcare professionals to work in rural areas of the US, which could further strengthen the workforce and enhance the delivery of IPC services [26].

The responses related to culturally centered care largely overlooked the needs of clients from Latino backgrounds, even though this population is included in the facility’s cultural competency training and is a priority population for public health research in northern Arizona [4]. This pattern may be due to the primary racial composition of clients who visit the center, mostly White and Native American individuals, which reflects the two largest racial groups in rural Arizona [4,20]. This client demographic likely influenced participants’ responses. It is also relevant that responses largely overlooked culturally centered care for clients with intersecting underserved identities. The human experience is shaped by factors such as race, class, immigration status, gender identity, and sexual orientation [27]. These identities do not exist in isolation; rather, they intersect with broader sociopolitical forces throughout minoritized people’s life course, such as societal hegemonies and historical time, to shape exposure to chronic stress related to hostility towards their identities and limit access to resources [27]. This temporal interaction, known as temporal intersectional minority stress, helps elucidate the disproportionate burden of SU/SUDs among individuals who hold intersecting underserved identities, as they may turn to substances as a means of coping with hostile or inequitable environments [27]. Recognizing the impact of intersectionality is thus crucial for ensuring that IPC is responsive to the unique needs of diverse clients with SU/SUDs.

Literature on behavioral health and primary care integration for LGBTQ+ communities and Native Americans demonstrates that integrated care holds strong potential to reduce disparities in healthcare access and outcomes. Yet, this potential can only be realized when integrated care services adopt interventions that are culturally responsive and aligned with community needs [28,29,30]. For instance, similarly to our findings, a systematic review identified that integrating community-based, traditional Native American knowledge and community representation into integrated care is important to strengthen care delivery approaches that address individuals’ overall well-being and that are sustainable [31]. Offering regular cultural competency training plays a central role in preparing IPC team members to care for diverse populations. However, meaningful change requires evaluating the quality and the outcomes of such training, ensuring that providers are prepared to care for those most impacted by SU/SUDs. In rural settings, this may involve collaborations with local advocacy organizations, hiring providers from underserved communities, and establishing community health advisory groups to guide culturally responsive care [31]. Additionally, accountability mechanisms must be in place to prevent discrimination and ensure equitable treatment, though this may be challenging due to evolving sociopolitical contexts [32].

Universities also have a critical responsibility in equipping future healthcare professionals with the skills to serve diverse populations by integrating cultural humility and social justice frameworks into healthcare education [33,34]. Equally important is advancing culturally centered research on SU/SUDs and training future scientists to conduct research that addresses systemic inequities [21]. By embedding these principles into education, research, and clinical practice, we can work toward a more inclusive and effective healthcare system.

One of the goals of integrated care models such as IPC is to strike a balance between health and social needs by engaging community assets to promote the overall well-being of individuals and communities [9]. In this study, we found that community partnerships enhance IPC by linking clients with SU/SUDs to essential resources beyond the facility’s offerings, such as housing, food assistance, peer support programs, and affordable medications, thereby addressing broader social determinants of health. Such partnerships help IPC to ensure that care delivery processes are meaningfully aligned with the health and social needs of those accessing care [35].

In addition to community partnerships, professional development, educational, and clinical initiatives can potentially enhance IPC’s capacity to support the health and social needs of clients with SU/SUDs. Interdisciplinary education sessions carried out with current providers have been shown to strengthen competencies to support clients’ health and social needs, as well as client outcomes [36]. For instance, clients seen by clinicians who participated in the CoEPCE (Centers of Excellence in Primary Care Education) [31] interdisciplinary training program had better outcomes than clients seen by clinicians who did not receive this training [32]. Clients seen by CoEPCE-trained providers had better diabetes management, more consistent preventive testing, safer prescribing practices for older adults, more timely mental health referrals, and fewer hospitalizations [37]. Mentorship and experiential learning can further develop professional development in IPC. The Early Career Researchers and Professionals in Integrated Care program, for example, is a one-year mentorship program that supports the next generation of integrated care stakeholders by enabling early career providers and researchers to receive mentorship from experienced professionals in integrated care models, offering mentees opportunities to gain practical experience in the field through research, internships and job placements [38]. Additional strategies for advancing integrated behavioral health and primary care are reported somewhere else and could serve as a resource for facilities implementing IPC [39,40,41]. Interdisciplinary care training should also be integrated into healthcare degree programs to prepare future healthcare professionals to IPC delivery. Simulation-based learning can be an approach to train students from diverse backgrounds (e.g., social work, medicine, and nursing) in comprehensively caring for people with SU/SUDs in a controlled environment [42,43].

### 4.1. Limitations and Strengths

This study should be interpreted in light of its limitations. The primary limitation, common to research in smaller communities with limited health services, is the small sample size, which restricts generalizability. The cross-sectional, single-site design further limits generalizability to other behavioral health centers implementing IPC in rural Arizona or similar regions in the US. Findings regarding stigma reduction and preventive care access as benefits of IPC are based on staff perceptions rather than client outcomes.

Potential selection bias is another limitation, as we did not use a sampling frame or role stratification for participant recruitment. However, the final sample included professionals from varied training backgrounds, providing a range of perspectives on IPC implementation. Social desirability bias may have influenced participants’ willingness to share honest opinions, although negative perceptions about service delivery were also reported. Full access to staff and providers allowed participants to speak openly. Potential power dynamics were mitigated by limiting facility representatives’ involvement to facilitating access and providing contextual input. Student engagement and reaffirming anonymity and voluntary participation further supported candid responses, including critiques of the center, highlighting the facility’s commitment to staff well-being and quality care.

We applied the SDH Framework to contextualize the study, but it was not used to guide the interview guide, coding framework, or theme development. Using the framework in these aspects could have strengthened our ability to systematically examine how IPC integrates community resources to support clients with SU/SUDs. Finally, the lack of intersectional analysis limits insights into culturally centered care. Although participants described care for diverse subpopulations (e.g., Native American and LGBTQ+ clients), data and illustrative quotes were insufficient to examine overlapping identities (e.g., LGBTQ+ Native Americans) or divergent experiences within these groups. As a result, findings may not fully capture the complexity of intersecting social identities, and caution is warranted in interpretation.

Despite these limitations, the depth of the interviews strengthens the study by capturing nuanced experiences from diverse staff that may inform similarly sized communities and health settings. The behavioral health center where the study was conducted is one of the few in the region offering IPC services, making this research essential for improving and expanding services for individuals with SU/SUDs.

### 4.2. Directions for Future Research

Building on participants’ perspectives on the implementation of IPC services in a rural Arizona behavioral health center, future research is warranted to optimize implementation, strengthen culturally centered care, and evaluate client outcomes of IPC services. Table 2 offers guidance for future research to strengthen IPC services and their responsiveness to diverse clients with SU/SUDs. Adopting a community-engaged research approach will be essential to ensure that future studies align with community priorities. Expanding research to include multiple behavioral health centers that also provide primary care services can further clarify how IPC models operate to comprehensively support rural Arizona residents with SU/SUDs.

While these recommendations are informed by a single-site study, they may also help guide studies on IPC implementation conducted at other behavioral health centers in rural settings.

## 5. Conclusions

This study addressed a gap in IPC research in the US by exploring healthcare providers’ perspectives on integrating primary care into a behavioral health center in northern Arizona. We found that IPC facilitates SU/SUD recovery by delivering holistic care that integrates medical, mental health, and addiction services while addressing clients’ social and co-occurring needs. It creates an empathetic environment in which clients are not required to repeatedly disclose their SU/SUD, enhances access to preventive care, and provides ongoing support and education. Implementation challenges included workforce shortages, limited internal communication, and insufficient interdisciplinary training. Gaps in culturally centered care were also noted, such as reliance on Western treatment models, underrepresentation of Native American and sexual and gender minority staff, and inconsistent use of inclusive practices like pronouns, traditional healing methods, and trauma-informed approaches. Furthermore, partnerships with multisectoral community organizations help clients access supportive resources beyond the facility, including vision care, clothing, and dental services.

Recommendations are provided for enhancing IPC to address the health and social needs of individuals with SU/SUDs in northern Arizona, including resources to support professional development in interdisciplinary care. Guidance for future research is also offered to strengthen IPC in Northern Arizona.

## Figures and Tables

**Table 1:** Interview guide for healthcare providers and administrative staff.

What is your role at this facility? What does integrated substance use disorders (SUDs) care and primary care mean to you? Do you believe providing primary care is important for patients experiencing SUDs? If yes, could you please explain why? In your opinion, what cultural practices should providers consider in primary and SUDs care integration? What resources are needed at [this behavioral health center] to effectively integrate primary care into SUDs care or vice versa? In your opinion, are there any difficulties in providing/implementing integrated primary care and SUDs care at [this behavioral health center]? If yes, please tell me more about these difficulties. Tell me if other organizations collaborate with [this behavioral health center] in the integration of care (i.e., dental care or other specialized care)

**Table 2:** Directions for future research on IPC implementation in a behavioral health center in northern Arizona.

**Internal Communication and Care Coordination:** Research should assess the frequency and effectiveness of interdisciplinary collaboration between providers at the behavioral health center (e.g., regular team meetings, shared patient records) and examine correlations with patient satisfaction and care quality metrics.
**Staff Training and Implementation of Best Practices in Interdisciplinary Care:** Research should evaluate interdisciplinary care training programs utilizing pre- and post- competency assessments, application of skills in practice, and associations with client satisfaction levels and health outcomes.
**Assessment of Healthcare Students’ Competency in Integrated Care for Clients with SU/SUDs:** Considering that the behavioral health center serves as clinical rotation site for university students in healthcare degree programs (e.g., nursing and social work), future research should evaluate the readiness of students in healthcare fields to collaborate in providing care that addresses the physical, mental, and social needs of clients with SU/SUDs in rural Arizona. Findings could inform curriculum development, interdisciplinary training initiatives, and strategies to better prepare future providers for IPC delivery within rural contexts.
**Provider Retention and Recruitment:** Quantitative evaluations of workforce stability are needed, including turnover rates, average tenure, and the influence of factors such as salary, workload, and workplace culture on staff retention.
**Access to Preventive Care:** Studies should assess whether IPC improves uptake of preventive services (e.g., screenings for liver disease, diabetes, and HIV and other sexually transmitted infections) among clients with SU/SUDs compared with behavioral health settings in rural Arizona, which do not offer IPC services. Assessing electronic health record data can be a valuable source of information for such research areas, including objective metrics on service utilization, disparities in access to care, and gaps in preventive care utilization.
**Stigma Reduction:** Future work should quantify reductions in stigma and improvements in patient self-worth associated with IPC, using validated patient-reported measures to capture changes over time. Researchers are encouraged to draw on the instrument inventory synthesized by a recent systematic review of measures of substance use-related stigma [44].
**Client Health Outcomes:** Future studies should measure the impact of IPC on patient-level health outcomes, including reductions in emergency department visits, improvements in physical health indicators (e.g., BMI, blood pressure), and management of comorbid conditions.
**Community Partnerships and Referrals:** Future studies should evaluate the role of partnerships with other organizations in rural Arizona to support clients’ health and social needs, tracking referral volumes, utilization of external services (e.g., dental, housing, emergency care), associations with recovery outcomes such as sobriety duration, and availability of resources to facilitate engagement with external organizations (e.g., transportation). Mixed-methods research on client referrals across health system levels underscores the importance of implementing robust client referral systems and can inform these studies [45].
**Culturally Centered Care:** Research should examine the effectiveness of cultural competency training, linking provider practices to satisfaction, adherence to care, and health outcomes among clients with SU/SUDs. Quantification of adherence to culturally centered practices (e.g., preferred pronouns, incorporation of traditional healing) would provide insight into gaps and opportunities for improvement. Additionally, it is crucial that future research explore culturally centered care based on an intersectional lens (e.g., healthcare for LGBTQ+ Native Americans) to ensure that care delivery is responsive to the complex health and social needs of diverse clients. Future studies should also assess culturally centered care for clients who belong to Latino populations and associated client outcomes to help inform interventions that promote intersectional-aware, culturally centered care.
**Utilization of Implementation Science Frameworks:** Future research should more fully engage with qualitative methods literature on organizational change and implementation, using resources such as the Social Care Implementation Framework [46], to move beyond descriptive reporting and deepen analytical insights.
**Comprehensive IPC Evaluation:** Longitudinal studies are needed to assess overall IPC implementation barriers and benefits, including cost-effectiveness, improvements in patient health outcomes, and facility performance indicators such as wait times and no-show rates.

## Data Availability

The original contributions presented in this study are included in the article. Further inquiries can be directed to the corresponding author.
